# Mechanisims of asthma and allergic disease – 1071. Chemokines from cord blood mononuclear cells and the development of eczema or wheeze at 2 years of age in infants at high risk for atopy

**DOI:** 10.1186/1939-4551-6-S1-P68

**Published:** 2013-04-23

**Authors:** Phaik Ling Quah, Chiung-Hui Huang, Lynette,Pei Shi Shek, Marion Aw, Bee Wah Lee, I-Chun Kuo

**Affiliations:** 1 Paediatrics, National University of Singapore, Singapore; 2 The Child & Allergy Clinic, Singapore

## Background

Innate chemokines secreted from cord blood mononuclear cells (CBMCs) offer novel tools to investigate the influence of immune deviation at birth to the development of eczema or wheeze by 2 years of age.

## Methods

Cord blood samples were collected from 161 subjects from a birth cohort of 253 subjects participating in a double-blind placebo randomized trial on probiotic supplementation (*Lactobacillus rhamnosus* GG and *Bifidobacteria longum*, birth to 6 months). Clinical symptoms for eczema and wheeze were followed up to 2 years of age. The chemokine production in culture supernatants from CBMCs stimulated with 1 ug/mL of lipopolysaccharides (LPS) for 24 hours was measured using the Milliplex assay. Chemokine levels were analyzed by the Mann-Whitney *U*Test and the association of chemokines with clinical outcomes was analyzed by the multivariable logistic regression.

## Results

Our results showed that subjects with eczema (n = 36) had significantly reduced production of CXCL5 (epithelial neutrophil-activating protein 78 (ENA78)) from CBMCs as compared to healthy control subjects (n = 94) from the LPS stimulated cells (*p* = 0.001). CCL20 (macrophage inflammatory protein-3α) levels were significantly lower in the subjects who developed wheeze (n = 35) as compared to healthy controls (n = 94) from the LPS stimulated cells (*p* = 0.001). There was no difference in CXCL5 and CCL20 production from the cultured unstimulated cells. Diminished CXCL5 production by LPS stimulated CBMCs was found to be independently associated with eczema (adjusted OR, 0.275; 95% CI, 0.134-0.562, *p* = 0.001) after adjusting for allergen sensitization, paternal eczema and sibling eczema using multivariable logistic regression. Similarly, suppressed production of LPS stimulated CCL20 was an independent risk factor associated to the development of wheeze (adjusted OR, 0.308; 95% CI, 0.133-0.716, *p*= 0.06) after adjustment for maternal asthma, sibling eczema, birth height and birth weight.

## Conclusions

We observed the diminished production of CXCL5 and CCL20 by CBMCs from subjects who develop eczema and wheeze respectively within 2 years of age. This association may reflect a deviated innate immune response to microbial or viral infections which might have an impact on the susceptibility of childhood eczema and wheeze.

